# Late-Onset Rheumatoid Arthritis (LORA): A Diagnostic and Therapeutic Challenge Among Older Patients Visiting a Poorly Resourced Health-Care Setting

**DOI:** 10.31138/mjr.29084.ada

**Published:** 2024-12-31

**Authors:** Ujjwol Prasad Risal, Urza Bhattarai

**Affiliations:** Department of Internal Medicine, B. P. Koirala Institute of Health Sciences, Dharan, Nepal

**Keywords:** late-onset rheumatoid arthritis (LORA), older patients, diagnostic and therapeutic challenge

## Abstract

Late-onset rheumatoid arthritis (LORA) presents a unique diagnostic challenge among older patients, particularly in poorly resourced healthcare settings. As global life expectancy increases, so does the prevalence of LORA, a condition that differs significantly from young-onset rheumatoid arthritis (YORA). This review explores the distinct clinical presentation, differential diagnosis, laboratory findings, and treatment challenges of LORA, emphasising its impact on low- and middle-income countries. The atypical and often acute onset of LORA, coupled with limited access to healthcare and diagnostic tools, contributes to significant diagnostic delays. These delays are compounded by a scarcity of healthcare providers, particularly rheumatologists, and the lack of essential laboratory tests in remote areas. Moreover, older adults often face additional barriers, including poor social support, reluctance to use allopathic medicines, and non-compliance with follow-ups. Effective management of LORA requires not only an understanding of its unique characteristics but also a tailored approach that considers the constraints of resource-limited settings. This review highlights the urgent need for specific guidelines and strategies to improve the diagnosis and management of LORA, thereby addressing the growing healthcare needs of older population in LMICs.

## INTRODUCTION

Currently, 11% of the world's population is over the age of 60 years with a projected increase to 22% by the year 2050.^1^ The rise in life expectancy is accompanied by a surge in age-related morbidities, particularly musculo-skeletal conditions like joint aches and pains, which are often underdiagnosed, especially in low- and middle-income countries (LMICs). This underdiagnosis is coupled with significant psychological stress and healthcare utilisation costs, exacerbating the burden on healthcare systems.^2^ Furthermore, among older adults, the clinical presentation of diseases is atypical, which adds to the diagnostic dilemma and presents therapeutic challenges.

Late-onset rheumatoid arthritis (LORA) refers to rheumatoid arthritis (RA) with an onset of disease activity occurring in old age. It is a condition that differs from typical RA which primarily affects middle-aged individuals. LORA is more challenging to diagnose and manage due to its atypical presentation. This demands a more nuanced understanding of the disease, particularly in resource-limited settings where diagnostic tools are often scarce.

The objective of this review is to provide an updated, in-depth analysis of the clinical presentation, differential diagnosis, laboratory findings, and treatment challenges of LORA, with a focus on its impact in LMICs, such as Nepal. In doing so, we hope to assist healthcare providers in overcoming the diagnostic and therapeutic challenges posed by LORA in the aging population. This review incorporates recent evidence and expands on previously published research, aiming to fill gaps in understanding the disease's unique characteristics in low-resource environments.

## METHODOLOGY

A comprehensive literature search was conducted to gather relevant studies on LORA with a particular focus on its clinical presentation, differential diagnosis, laboratory findings, and treatment in low-income countries. The search was conducted using three major databases: Medline/PubMed, Scopus, and the Directory of Open Access Journals (DOAJ). The search covered latest studies on LORA and was conducted following the recommendations provided in the Preferred Reporting Items for Systematic Reviews and Meta-Analyses (PRISMA) guidelines. Specific search terms and Medical Subject Headings (MeSH) included “Late-onset rheumatoid arthritis,” “LORA,” “elderly onset rheumatoid arthritis,” “EORA”, “rheumatoid arthritis in the elderly,” “clinical presentation,” “differential diagnosis,” and “low-income countries.”

## DEFINITION AND EPIDEMIOLOGY

Late-onset rheumatoid arthritis (LORA) is also referred to as elderly-onset rheumatoid arthritis (EORA). The exact age cut-off for the onset is still a matter of debate. However, the onset of RA after the age of 60 or 65 years has been generally accepted in the medical literature.^3,4^ The prevalence of RA globally has been estimated to be 0.24 % but is as high as 1 % in Western countries.^5,6^ Similarly, the prevalence in LMICs has been found to range from 0.37 to 1.25%.^7^ Studies have shown that about one-third of RA cases develop in patients over the age of 60 and the prevalence increases with age.^8^ There is a paucity of data regarding the prevalence of LORA worldwide. One study from the USA has shown that the prevalence of RA over the age of 60 years is around 2%.^9^ According to the Global Burden of Disease Study 2019, 55 % of patients with RA are older than 55 years.^10^ Based on the above data, it can be estimated that LORA makes up a significant proportion of RA patients.

## PATHOGENESIS

The exact cause of LORA is unknown and is an area of ongoing research. Similar to young-onset RA (YORA), various genetic, environmental and immunological factors may be implicated in the causation of the disease.^11^ Genetic studies regarding the role of HLA -DRB1 gene have been inconclusive.^12,13^ However, there are certain physiological changes in the immune system with aging that may play a role in the development of LORA. The aging immune system loses the ability to fight against infections and cancer, and has a diminished response to vaccination but makes the individual susceptible to inflammatory disorders.^14^ Immunosenescence is characterised by short-lived memory responses, defective response to new antigens, higher predisposition to autoimmunity, and chronic low grade inflammation aptly named inflammaging.^15^ Some investigators have linked the role of interleukin -6 (IL-6) with the causation of LORA based on increased synovial fluid IL-6 levels in patients with LORA in comparison to those with YORA.^16^ Another study has also suggested the role of IL-6 which increases secondary to a decline in sex hormones such as dehydroepiandrosterone (DHEA), DHEA sulphate (DHEAS) and androstenedione with aging.^17^ In summary, the aging immune system plays a significant role in the causation and propagation of the disease.

## CLINICAL PRESENTATION

The clinical presentation of LORA is quite distinct from YORA. YORA predominantly affects females in a ratio of 3:1, but LORA affects both males and females equally.^18^ YORA most commonly has a subacute or chronic onset, whereas LORA usually has an acute onset which resembles viral arthritides and may cause a delay in diagnosis.^8^ The pattern of joint involvement in YORA is usually bilaterally symmetrical polyarthritis predominantly involving the small joints of hands and feet. LORA may have three patterns of joint involvement. 70% of patients have a similar presentation to classic RA with positive rheumatoid factor (RF), joint erosions and a worse prognosis than YORA. The second presentation is similar to polymyalgia rheumatica (PMR) which is seen in about 25 %. Patients usually are RF negative and have a better prognosis. The third presentation is similar to remitting seronegative symmetrical synovitis with pitting oedema (RS3PE) syndrome.^4^ RS3PE syndrome is characterised by an acute onset polyarthritis, pitting oedema of hands, male predominance, elevated acute phase reactants and an excellent prognosis.^4,6^ Systemic manifestations like fatigue and weight loss are more prominent in LORA than YORA.^8,18^

### Differential diagnosis

Since the presentation of LORA is acute, viral arthritis is an important differential diagnosis. Paraneoplastic arthritis is another important differential diagnosis, as malignancy is more common among older adults. PMR and RS3PE syndrome, polyarticular gout, pseudogout, erosive hand osteoarthritis are other important differentials that need to be ruled out.

### LORA and geriatric syndromes

Older patients with LORA and multiple co-morbidities typically have numerous geriatric syndromes that interfere with their basic daily activities and quality of life. Pain and impaired mobility contribute to unsteadiness and an increased risk of fall and fall related injuries. Multiple chronic conditions and age-unfriendly rural infrastructure contribute to incidences of fall especially in low-income households.^19^ Disease burden, social isolation and functional decline result in psychological issues and depression. Chronic low-grade inflammation is associated with frailty resulting in muscle weakness, slow walking speed and unintentional weight loss.^20^ Polypharmacy and inflammation associated with disease burden contribute to anorexia and cognitive impairment.^21^ Worldwide, and especially in LMICs, there is a widespread prevalence of food-related myths and misconceptions associated with rheumatological conditions. These misconceptions and disease related factors contribute to undernutrition among older adults.^22^ It has been reported that, compared with subjects without geriatric syndromes, older RA patients with geriatric syndromes have a longer disease duration, higher DAS28 scores, lower haemoglobin levels, and greater physical dependence.^23^ Therefore, the chief complaints of patients with LORA may not represent a specific pathological condition but may be related to multiple underlying factors involving multiple organ systems and issues. These geriatric syndromes must be taken into consideration during the comprehensive evaluation of older patients with LORA and complex multimorbidity.

### Laboratory abnormalities

Since most patients with LORA have an acute onset with intense inflammation, they tend to have highly raised inflammatory markers, such as erythrocyte sedimentation rate (ESR), C-reactive protein (CRP), and a higher degree of anaemia in comparison to patients with YORA.^24^ Even though earlier studies had shown a decreased prevalence of rheumatoid factor (RF) in patients with LORA, newer studies have not found significant differences in terms of RF and anti-citrullinated protein antibody (ACPA) positivity.^25,26^

### Challenges in a low-resourced setting

Low-resource settings, including LMICs often have difficult geographical and infrastructural barriers that delay access to healthcare services. Access to basic healthcare settings is challenging, with only a portion of the population able to reach health services within a reasonable timeframe. For example, in some regions, only about 62% of the population may have access to healthcare within 30 minutes.^27^ Additionally, significant disparities exist between urban and rural populations in these settings, where doctor-to-patient ratios can be as skewed as 1:850 in urban centres versus 1:150,000 in rural areas.^28^ The disproportionate distribution of health-care personnel and the inaccessibility of rural and remote regions contribute to increased morbidity among older adults with RA. Moreover, the availability of specialised care, such as rheumatology services, is often limited, with most specialists concentrated in tertiary care referral hospitals. This makes it essential for healthcare providers in remote or under-resourced regions to recognise the atypical presentations of RA in older adults, helping to avoid underdiagnosis and poor treatment outcomes in this vulnerable population.

In rural and low-resource settings, access to essential laboratory tests, including CRP, RF, and ACPA, is often limited. This shortage of critical diagnostic tools significantly impedes the accurate and timely diagnosis of rheumatological disorders, posing a major challenge for healthcare providers in these areas.

Delays in diagnosis further complicate proper management of LORA in these settings. These delays are caused by not only under-resourced healthcare systems and limited manpower but also by various patient-related factors. Patients with low socioeconomic backgrounds present late with high disease activity, resulting in significant joint damage and deformities that are difficult to reverse. In addition, there may be reluctance to use allopathic treatments, with many patients favouring herbal preparations or local treatments. The rapid migration of younger populations to urban centres or abroad also reduces the availability of caregivers for the elderly. As family structures evolve and caregiving resources dwindle, elderly individuals with arthritis are often neglected, delaying their access to appropriate rheumatological care. Patients treated with disease-modifying anti-rheumatic drugs (DMARDs) require frequent follow-up visits and tests. However, older adults living in low-resource settings often struggle with adherence due to insufficient social support systems.

These challenges have been summarised in **Table 1**, and categorised into healthcare system-related challenges, diagnostic challenges, and patient-related challenges.

**Table 1.** Challenges related to health care system, diagnostic challenges, and patient-related challenges.

	**Key challenges**
**Health care system-related challenges**	Geographical constraints and topographical difficulties to reach health care facility Low doctor-patient ratio Handful of rheumatologists Rapid out-migration of healthcare professionals
**Diagnostic challenges**	Lack of essential laboratory tests Expensive laboratory tests
**Patient-related challenges**	Poor social support among the elderly due to the rapid outmigration of youth in the family Late health-care-seeking behavior Reluctance to use allopathic medicine Use of herbal and traditional healers Non-compliance with follow-ups and tests advised

## TREATMENT

Even though the basic principles of treatment for YORA apply to LORA as well, there are many factors that need to be considered in these vulnerable patients. Older adults have different pharmacokinetics and pharmacodynamics compared to younger individuals.^29^ By the time they are diagnosed with LORA, they often already have multiple comorbidities, such as hypertension, diabetes, cardiovascular and lung diseases, chronic kidney disease, malignancy, osteoarthritis, neuropsychiatric disorders, and osteoporosis, for which they are on multiple medications.^30^ Therefore, it is highly likely that medications used to treat LORA may interact with these other drugs, leading to unpredictable responses and unwanted adverse effects, including severe drug toxicity. There is also a risk of forgetting to take prescribed medications due to the high pill burden and cognitive decline, such as dementia, especially in the absence of caregivers. Patients with LORA are particularly vulnerable to the adverse effects of non-steroidal anti-inflammatory drugs (NSAIDs) which can lead to gastrointestinal (GI) bleeding, peptic ulcers, and erosion.^31^ Studies have shown that age itself is a risk factor for GI bleeding, and the use of NSAIDs increases this risk four-fold.^32^ Similarly, the risk of acute kidney injury and cardiotoxicity is higher among older adults with the use of NSAIDs.^33,34^

Steroid therapy has been found to be highly effective as a bridging therapy in patients with RA and may also be applicable to those with LORA.^35,36^ However, there are many challenges associated with the use of steroid therapy in these patients, especially in LMICS. Studies have shown that older patients are more prone to the adverse effects of steroid therapy, such as hypertension, osteoporosis, and hyperkalemia.^37^ In LMICs, steroids are among the most commonly used over-the-counter drugs due to their low cost, easy availability, and the lack of prescription requirements for their sale.^38,39^ Once patients experience symptomatic improvement from steroids, they often become dependent on them and tend to use them for years. This results in various harmful effects associated with long-term steroid use.

Low-dose methotrexate, taken once a week with folic acid supplementation, is the cornerstone of therapy in the management of RA and has been found to have similar efficacy and safety profile in LORA.^40,41^ However, older adults may mistakenly take it daily, leading to potentially fatal methotrexate toxicity. One of the reasons for this is illiteracy among this group of people in LMICs.^42^

Additionally, they are more vulnerable to the adverse effects of hydroxychloroquine compared to younger patients because of a decline in renal function and drug interactions leading to retinal and cardiovascular toxicity.^43,44^ Sulfasalazine and leflunomide are easily available and have been shown to have similar safety profile in patients with LORA.^45^ All the above DMARDs need baseline evaluation and frequent monitoring for disease activity and drug toxicity.^46^ However, many stop taking DMARDs once they become symptom-free, some continue using DMARDs for an extended period without follow-up, while others self-medicate with steroids alone.

The use of newer drugs, such as biologics, biosimilars, and targeted synthetic DMARDs, remains in its early stages due to limited access, high costs, lack of education, insufficient monitoring, a high prevalence of tuberculosis and other infections, and an inadequate vaccination coverage.

## CONCLUSIONS

The mode of presentation, laboratory tests, and treatment approach differ in patients with LORA. Additionally, recognising and treating these patients, particularly in resource-limited settings, presents many challenges. When preparing guidelines, rheumatology societies should include a separate section on the management of LORA.

## Data Availability

Not applicable.
